# Transcranial Direct Current Stimulation (tDCS) Can Modulate EEG Complexity of Children With Autism Spectrum Disorder

**DOI:** 10.3389/fnins.2018.00201

**Published:** 2018-04-16

**Authors:** Jiannan Kang, Erjuan Cai, Junxia Han, Zhen Tong, Xin Li, Estate M. Sokhadze, Manuel F. Casanova, Gaoxiang Ouyang, Xiaoli Li

**Affiliations:** ^1^Institute of Electrical Engineering, Yanshan University, Qinhuangdao, China; ^2^Institute of Biomedical Engineering, Yanshan University, Qinhuangdao, China; ^3^State Key Laboratory of Cognitive Neuroscience and Learning, IDG/McGovern Institute for Brain Research, Beijing Normal University, Beijing, China; ^4^Key Laboratory of Measurement Technology and Instrumentation of Hebei Province, Qinhuangdao, China; ^5^Department of Biomedical Sciences, School of Medicine Greenville Campus, Greenville Health System, University of South Carolina, Greenville, SC, United States

**Keywords:** autism spectrum disorder (ASD), transcranial direct current stimulation (tDCS), electroencephalography (EEG), complexity, maximum entropy ratio (MER)

## Abstract

Autism spectrum disorder (ASD) is a heterogeneous neurodevelopmental disorder which affects the developmental trajectory in several behavioral domains, including impairments of social communication, cognitive and language abilities. Transcranial direct current stimulation (tDCS) is a non-invasive brain stimulation technique, and it was used for modulating the brain disorders. In this paper, we enrolled 13 ASD children (11 males and 2 females; mean ± SD age: 6.5 ± 1.7 years) to participate in our trial. Each patient received 10 treatments over the dorsolateral prefrontal cortex (DLPFC) once every 2 days. Also, we enrolled 13 ASD children (11 males and 2 females; mean ± SD age: 6.3 ± 1.7 years) waiting to receive therapy as controls. A maximum entropy ratio (MER) method was adapted to measure the change of complexity of EEG series. It was found that the MER value significantly increased after tDCS. This study suggests that tDCS may be a helpful tool for the rehabilitation of children with ASD.

## Introduction

Autism spectrum disorder (ASD) is a neurodevelopmental disorder characterized by an impairment in social communication, restricted interests and stereotypical behaviors (Rapin, 1997). The estimated prevalence of ASD in America is 1 in 68, reflecting a nearly 30% rate increase within the last 2 years (Deborah et al., 2015). The causes and pathologic mechanisms of autism are still unclear (Trottier et al., 1999). Genetic studies have revealed that several single-gene disorders and rare copy number variants appear to be strongly associated with ASD; however, genetic syndromes, mutations, and single-gene etiologies account for only 10 to 20% of ASD cases, and in many cases individuals with these genetic syndromes do not have an ASD diagnosis (Abrahams and Geschwind, 2008). Recently, neuroimaging tools have been applied for the evaluation or assistant diagnosis of ASD. For examples, functional magnetic resonance imaging (fMRI) have demonstrated functional under-connectivity in ASD (Just et al., 2007) and reported abnormalities in individuals with ASD when performing tasks that include working memory and face recognition (Pierce et al., 2001; Koshino et al., 2008).

Some studies have demonstrated the imbalances between excitation and inhibition in synaptic transmission and neural circuits in autism spectrum disorders (Dickinson et al., 2016; Lee et al., 2016). Pathophysiological alterations in the glutamate (Glu) andγ-aminobutyric acid (GABA) metabolism might lead to an excitatory-inhibitory imbalance. Glu is the most important excitatory and GABA the most important inhibitory neurotransmitter in brain. Some authors have proposed the idea that such genetic changes lead to an imbalance of excitation and inhibition in cortical regions, which might be a critical cause correlate of autistic symptoms (Rubenstein and Merzenich, 2003; Rubenstein, 2010). Recent anatomic studies have shown that autism is closely associated with abnormalities in cortical minicolumns. The latter are reduced in size and increased in number in ASD, especially within the dorsolateral prefrontal cortex (DLPFC) (Casanova, 2006). This may cause a bias in the ratio of cortical excitation to inhibition, which adversely affects the functional distinctiveness of minicolumnar activation.

Transcranial direct current stimulation (tDCS) and transcranial magnetic stimulation (TMS) are non-invasive brain stimulation techniques that have yielded promising and encouraging outcomes for the treatment of psychiatric disorders. A number of clinical studies have reported positive outcome measures when using TMS as a therapeutic intervention for ASD (Casanova et al., 2014; Panerai et al., 2014; Sokhadze et al., 2014), and TMS is considered safe if applied within safety guideline (Rossi et al., 2009). tDCS provides a weak constant current, ranging from 1 to 2 mA to the scalp through two electrodes: an anode and a cathode (Nitsche et al., 2008). It can modulate the spontaneous neuronal activity by inducing either positive (anodal) or negative (cathode) intracranial current flow in specific brain regions. Anodal stimulation increases cortical excitability, whereas cathode stimulation inhibits the same (Nitsche and Paulus, 2000; Terney et al., 2008). These currents modify the transmembrane neuronal potential and thus influence the level of excitability thus modulating the firing rate of neurons in response to additional inputs (Wagner et al., 2007). In addition, anodal stimulation can induce a significant increase in the regional cerebral blood flow (rCBF) combined with functional near-infrared spectroscopy (fNIRS), which offers a measure of regional blood oxygenation state in cortical tissue and cerebral blood flow that could reflect neuronal activity (Merzagora et al., 2010). The effects of tDCS are associated with a number of different mechanisms, including local changes in ionic concentrations (hydrogen, calcium) and levels of cyclic adenosine monophosphate (cAMP), alterations in protein synthesis, and modulation of N-methyl-D-aspartate (NMDA) receptor efficacy (Islam et al., 1995; Nitsche et al., 2004; Merzagora et al., 2010). Magnetic resonance spectroscopy (MRS) has shown that anodal stimulation reduces local concentrations of the inhibitory neurotransmitter GABA, whereas cathodal stimulation reduces excitatory glutamate levels (Stagg et al., 2009; Clark et al., 2011). In this study, we try to demonstrate the effects of the tDCS on the ASD. In our study the dorsolateral prefrontal cortex was selected due to its important role in cognition. In addition, some studies have incriminated functional abnormalities in this cortical region as a contributor to the pathogenesis of autism and there is a relationship between social disability and metabolic dysfunction in this region (Fujii et al., 2010).

Electroencephalography (EEG) provides a precise millisecond-timescale temporal dynamics to measure the postsynaptic activity in the neocortex and is a powerful tool to study the complex neuropsychiatric disorders. Previous studies showed EEG changes in some brain regions of the children with autism. Some EEG analysis methods have been used to reveal these EEG changes in ASD during resting state or performing some specific tasks. Resting-state EEG studies of ASD suggest a U-shaped profile of electrophysiological power alterations, with excessive power in low-frequency and high-frequency bands (Wang et al., 2013). The EEG recordings have also been used for assessing functional connectivity between different brain regions and calculating the spectral power changes of ASD (Murias et al., 2007; Wang et al., 2013; Righi et al., 2014). In the context of entropy, a high entropy value implies the ability to store more information within a neural network. The recent nonlinear approaches characterizing complex temporal dynamics have provided new insights into EEG dynamic complexity in mental disorders including ASD. The multiscale entropy approach was used to measure the changes in EEG complexity for ASD after electroconvulsive therapy (Takahashi, 2013). Combining discrete wavelet transform (DWT), entropy (En) and artificial neural network (ANN) methods were used for assisting autism diagnosis (Djemal et al., 2017). Transfer entropy methods could reconstruct connectivity of simulated neuronal networks of both excitatory and inhibitory neurons (Orlandi et al., 2014). Maximum entropy ratio (MER) is a new symbolic analysis approach for the detection of recurrence domains of complex dynamical systems from time series (Graben and Hutt, 2013; Beim Graben and Hutt, 2015). The method has been successfully adapted to investigate the high-dimensional electrocorticogram (ECoG) data for epilepsy patients (Yan et al., 2016). In this paper, this method will be used to measure the changes of EEG of patients with ASD. Previous studies have shown that such an imbalance could cause low entropy neural network dynamics (Catarino et al., 2011; Okazaki et al., 2015), suggesting that the mechanism which causes low entropy in autistic brain circuits may be an imbalance of cortical excitation and inhibition. We aimed to investigate the EEG changes induced by anodal tDCS over the DLPFC during the resting state of ASD children and if it was effective to alter the excitatory and inhibitory imbalance. In this paper, a MER is applied for calculating and comparing pre- to post-tDCS changes of EEG data from a group of children with ASD who received 10 times anodal tDCS modulation over the left dorsolateral prefrontal cortex (F3 in the 10/20 international electrode placement system) and EEG data changes of non-modulation from another group of autistic children matched by gender and age who were waiting for the next experiments.

## Materials and methods

### Subjects

We studied 13 subjects (11 males and 2 females; mean ± SD age: 6.5 ± 1.7 years) who received 10 times tDCS modulation for 3 weeks and 13 subjects (11 males and 2 females; mean ± SD age: 6.3 ± 1.7 years) who waited for the experiment as controls in the current study. They were all diagnosed to be ASD by professional psychiatrists in China based on PEP-III (Chen et al., 2011) and DSM-IV-TR criteria (Sadler and Fulford, 2006). All participants provided written informed consent and their parents were informed of the whole experimental procedure before participation. The clinical trial was conducted in accordance with the Declaration of Helsinki and was approved by the Beijing Normal University ethics committee.

Study inclusion criteria include: (1) participants with autism; (2) age between 4 and 8 years. Study exclusion criteria included: (1) use of a pacemaker or other metal device in the body; (2) skull defects; and (3) having a diagnosis of epilepsy.

### tDCS stimulation

A direct current of 1 mA was delivered using a battery driven constant-current stimulator (neuroConn GmbH, Ehrenbergstr, Ilmenau, Germany). During stimulation the impedance value was maintained below 50 kΩ between two saline-soaked surface sponge electrodes (7 × 4.5 cm). The anodal electrode was placed over DLPFC and the cathode electrode was placed over the right supraorbital. In the stimulation session, the current was ramped up from 0 to 1 mA in 30 s. 20 min after onset, the current was ramped down back to 0 in 30 s. Every subject received 10 tDCS sessions once every other day.

### EEG recording and data analysis

EEG was recorded in a quiet room, with the subject being awake, seated on a comfortable chair and relaxed in an eyes-open state without other activities (such as shaking head, gritting teeth, or facial movement). In the process, we collected 5 min resting-state EEG data by 128 HydroCel Sensor Net System (Electrical Geodesics, Inc.), setting the central vertex as the referential electrode (impedances less than 50 K; sampling rate 1,000 Hz). We collected EEG data of every subject before the experiment as a baseline and again after conclusion of the trial. For controls, we collected EEG data once and again after 3 weeks. During the process, they did what they usually did. We selected 19 electrodes (the standard international 10–20 electrode placement: Fp1, Fp2, F3, F4, C3, C4, P3, P4, O1, O2, F7, F8, T7, T8, P7, P8, Fz, Cz, and Pz) for a more accurate result.

The recorded EEG data were analyzed offline by Net Station 4.5.2 software (Electrical Geodesics). First, we processed the raw data by independent components analysis (ICA) to reject some artifacts such as ocular and muscular artifact (eye blink, eye movement, generic discontinuity and electromyography) by visual inspection. An additional stop-band filter at 50 Hz was applied. Then the data were band-pass filtered between 0.5 and 65 Hz. The data were down sampled to 250 Hz. Afterward, the EEG signals were selected and dissected from pre-tDCS and post-tDCS states and from controls. For each dataset, a total of 50 five-second 19-channel EEG epochs were extracted. There were altogether 100 artifact-free EEG epochs. Afterwards, epoch rejection was based on both visual and computer selection. Thus, the EEG data, which were free from electrooculogram and movement artifacts, had minimal electromyographic activity.

### Maximum entropy ratio (MER)

The recurrence plot is a two-dimensional graphical representation which shows the periodic nature of some states (Webber and Zbilut, 1985). The MER method is obtained by transforming the traditional recurrence plot to a symbolic recurrence plot (Graben and Hutt, 2013). MER is a less time-consuming method and one more suitable for high-dimensional data since it simply exploits the recurrence structure of a system's dynamics. It needs only a few parameters but can process various complex signals with quantified results and statistical analysis (Beim Graben and Hutt, 2015).

Recurrence plot is used to visualize the time dependent behavior of orbits *x*_*i*_ in a phase space for calculating MER. The important step of a recurrence plot is to calculate the following *N* × *N* matrix:

(1) $$R_{i,j}=\{\begin{array}{c} 1:||x_{j}-x_{i}||\leq \varepsilon \\ 0:\;\text{Otherwise} \end{array}i,j\;=\;1,\ldots ,N$$

where *N* is the number of points in the times series for analysis, ||·|| is the norm (the *L*_∞_-norm is selected, because it is computationally faster and allows the study of some features in RPs analytically), ε is the cutoff distance defining an area centered at *x*_*i*_.

Then, a cluster list of RP results makes a new symbolic recurrence plot. The detailed steps are as follows:

Step 1: Define a recurrence plot of size *N* × *N*, and make a cluster for each row.
Step 2: Test if the cluster list is empty. If yes, the calculation ends. Else, create a new empty cluster.
Step 3: Choose one cluster from the cluster list which has not been compared with the new cluster. If the new cluster is empty, remove it from the cluster list.
Step 4: If the chosen cluster has the same index as new cluster, merge its indices into the new cluster and remove the cluster from the cluster list.
Step 5: Repeat steps 3–4 until all clusters in the cluster list have been compared. Save the new cluster into a new cluster list.
Step 6: Repeat steps 2–5 until the cluster list is empty.

Afterwards, a new cluster list is constructed, called a symbolic recurrence plot. The detailed process can be found in Yan et al. (2016).

Guided by the principle of maximal entropy, Graben et al. assumed that the system spends equal portions of time in its recurrence domains and derives a utility function of the symbolic encoding from the entropy of the symbol distribution (Graben and Hutt, 2013):

(2) $$\begin{array}{l} \text{H}\left(\varepsilon \right)=-\overset{\text{M}\left(\varepsilon \right)}{\underset{\text{k}}{\sum }}{\text{p}}_{\text{k}}\log{\text{p}}_{\text{k}} \end{array}$$

(3) $$\begin{array}{l} \text{h}\left(\varepsilon \right)=\frac{\text{H}\left(\varepsilon \right)}{\text{M}\left(\varepsilon \right)} \end{array}$$

where M(ε) is the cardinality of the cluster repertoire obtained for cutoff distance ε and *p*_*k*_ is the relative frequency of a new cluster; h(ε) is a good estimator for a given encoding, which is determined by ε. Small values of ε lead to an almost uniform distribution of rare clusters. Then, h(ε) is punished by the large alphabet. In contrast, large values of ε give rise to a trivial partition with small entropy. Thus, the quantity h(ε) assumes a global maximum for an optimal value:

(4) $$\begin{array}{l} \varepsilon^{*}={\text{max}}_{\varepsilon }\text{h}\left(\varepsilon \right) \end{array}$$

reflecting a uniform distribution of a small number of recurrence domains.

Finally, the MER is defined as:

(5) $$\begin{array}{l} \text{MER}=\text{h}\left(\varepsilon^{*}\right) \end{array}$$

The Shannon entropy H(ε) is different for different cardinality M(ε). As a simple example take the uniform distribution - it's maximized entropy is:

(6) $$\begin{array}{l} {\text{p}}_{\text{k}}=1/\text{M}\left(\varepsilon \right)\;\to \text{H}\left(\varepsilon \right)=\text{logM}\left(\varepsilon \right) \end{array}$$

Thus, for a given M(ε), the maximized entropy ratio is:

(7) $$\begin{array}{l} {\text{h}}^{\prime }\left(\varepsilon \right)=\log\text{M}\left(\varepsilon \right)/\text{M}\left(\varepsilon \right) \end{array}$$

**Figure 1:**
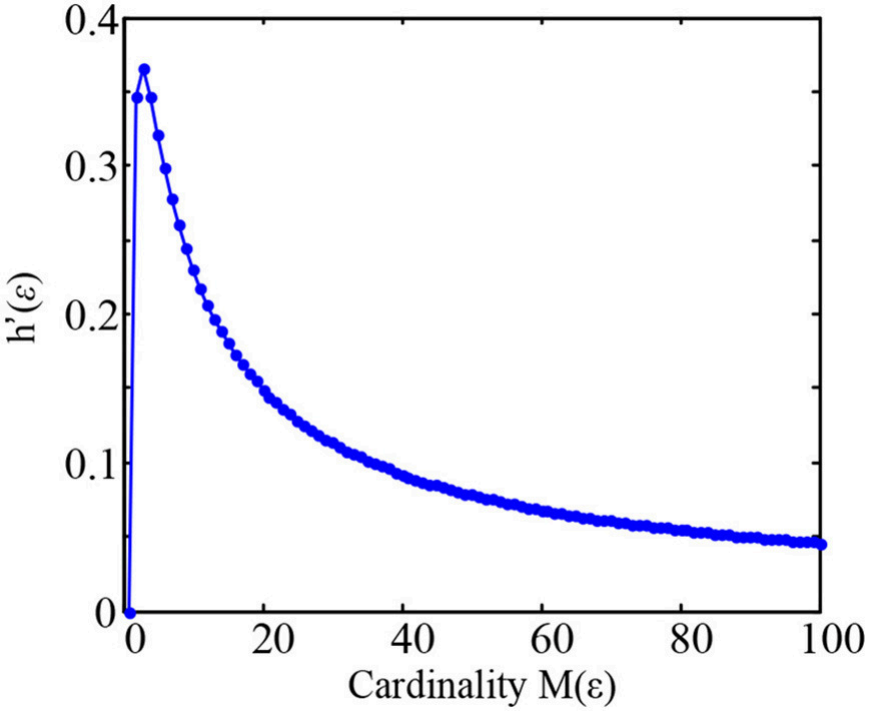
Plot of the maximized entropy ratio h′(ε) = log*M*(ε)/*M*(ε) as a function of M(ε) in the Equation (7).

For example, as the cardinality M(ε) increased from 1 to 100 and analytically computing h′(ε) as a function of M(ε) (Figure 1), it can be found that the value *h*′(ε) gradually increases and reaches its maximum at M(ε) = 3, and then gradually decreases at large cardinality M(ε).

So that the corresponding normalized maximum entropy ratio (NMER) is:

(8) $$\begin{array}{l} \text{NMER}=\text{h}\left(\varepsilon^{*}\right)/{\text{h}}^{\prime } \end{array}$$

### Software tools

MATLAB language and toolboxes were used for data processing and analysis (The Mathworks, USA). For the real execution Matlab function h(ε), the parameter ε start at 0.01 and gradually increased the value ε with 0.01 increment, it will stop execution until the value h(ε) is reach to zero.

## Results

We aimed to investigate whether tDCS had some effects on the EEG complexity of autistic children using the MER method. The results showed that the MER value was higher when comparing post-tDCS to pre-tDCS for experimental group and almost remained unchanged for controls, which means that the EEG complexity increased after one session of tDCS stimulation.

**Figure 2:**
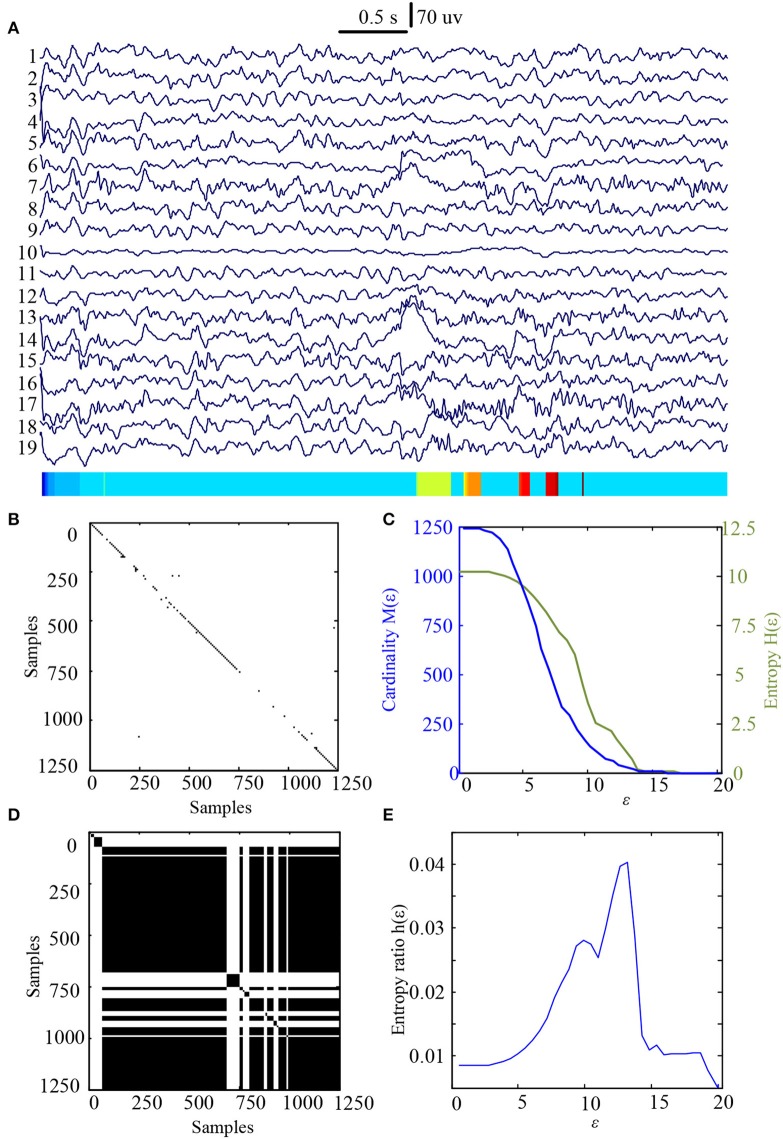
MER analysis of an EEG segment of pre-stimulation. **(A)** The original EEG data from the pre-tDCS state; **(B)** The recurrence plot of **(A)**, with ε = 13.2; **(C)** Change of entropy and cardinality with ε; **(D)** Symbolic recurrence plot; **(E)** Change of entropy ratio with ε (from 0.01 – 20.34 with 0.01 increment).

**Figure 3:**
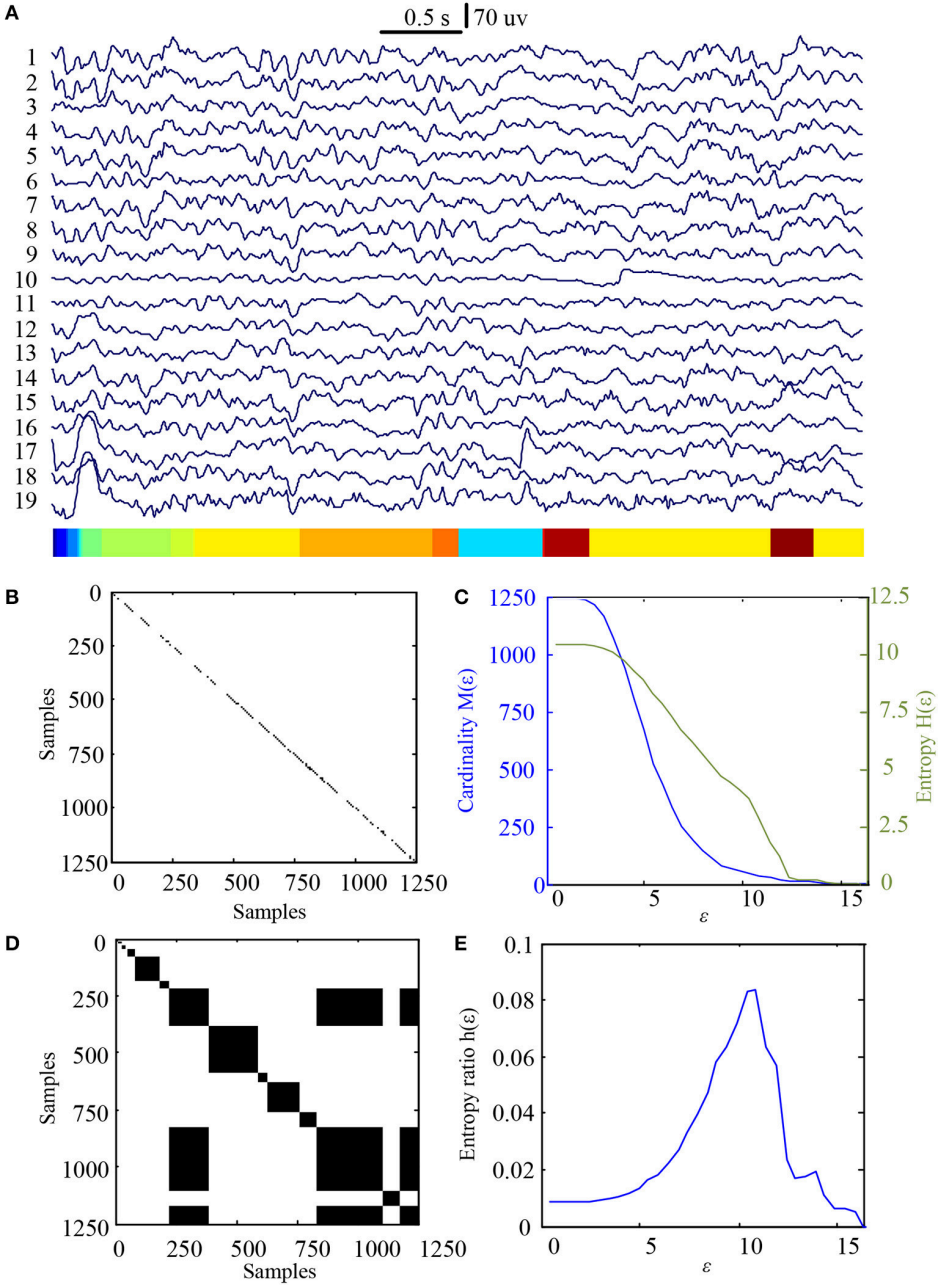
MER analysis of an EEG segment of post-stimulation. **(A)** The original EEG data from the post-tDCS state; **(B)** The recurrence plot of **(A)**, with ε = 10.88; **(C)** Change of entropy and cardinality with ε; **(D)** Symbolic recurrence plot; **(E)** Change of entropy ratio with ε (from 0.01 – 16.46 with 0.01 increment).

In this study 100 EEG epochs (50 from pre-tDCS and 50 from post-tDCS) from 13 ASD children were used for analysis. Each epoch's length is 5 s. The sample rate is 250 Hz. Figure 2B is the recurrence plot of the EEG data in Figure 2A, with ε = 13.2. We also calculate the entropy H(ε) and Cardinality M(ε) with the change of ε (Figures 2C,D) shows the symbolic recurrence plot using the MER algorithm, while Figure 2E shows the maximum entropy MER changing with ε, with a peak value of 0.0403 (correspondingly normalized maximum entropy NMER is 0.1100), and the optimal encoding ε^*^ is 13.2. The MER analysis result shown in Figure 3 is for a 5 s post-tDCS EEG. We calculated MER value based on the average of 19 channels which were chose for a more accurate result including Fp1, Fp2, F3, F4, C3, C4, P3, P4, O1, O2, F7, F8, T7, T8, P7, P8, Fz, Cz, and Pz. Every channel was given a value after calculation and we obtained the average result which showed the brain condition for autisitic children and the controls. The maximum entropy MER value is 0.0833, normalized maximum entropy NMER is 0.2275, and the optimal encoding ε^*^ is 10.88. The results showed that the normalized maximum entropy NMER value is 0.1100 before tDCS, and increased to 0.2275 after one session tDCS treatment and *t* test statistical method was used (*p* = 0.0259 < 0.05). The statistical results are listed in Table 1. MER was significant lower at the pre-tDCS state. On the other hand, the optimal encoding ε^*^ was within a lower range at the post-tDCS state.

**Table 1:** Statistic results of ε^*^ and NMER values with pre-tDCS and post-tDCS.

	**Pre-tDCS**	**Post-tDCS**
ε^*^	26.29 ± 21.39	11.13 ± 2.40
NMER	0.1144 ± 0.0415^*^	0.2105 ± 0.0664^*^

**Figure 4:**
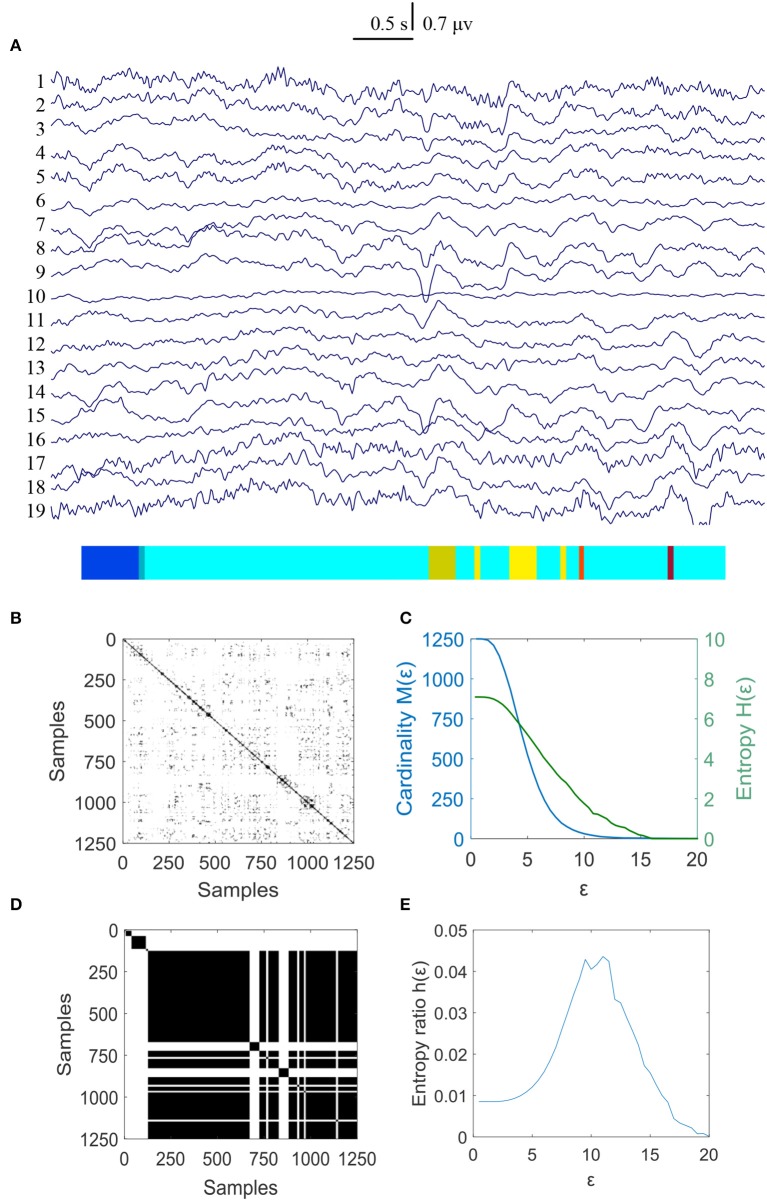
MER analysis of an EEG segment for the controls at the first time. **(A)** The original EEG data from controls; **(B)** The recurrence plot of **(A)**, with ε = 11.16; **(C)** Change of entropy and cardinality with ε; **(D)** Symbolic recurrence plot; **(E)** Change of entropy ratio with ε (from 0.01 – 20.00 with 0.01 increment).

**Figure 5:**
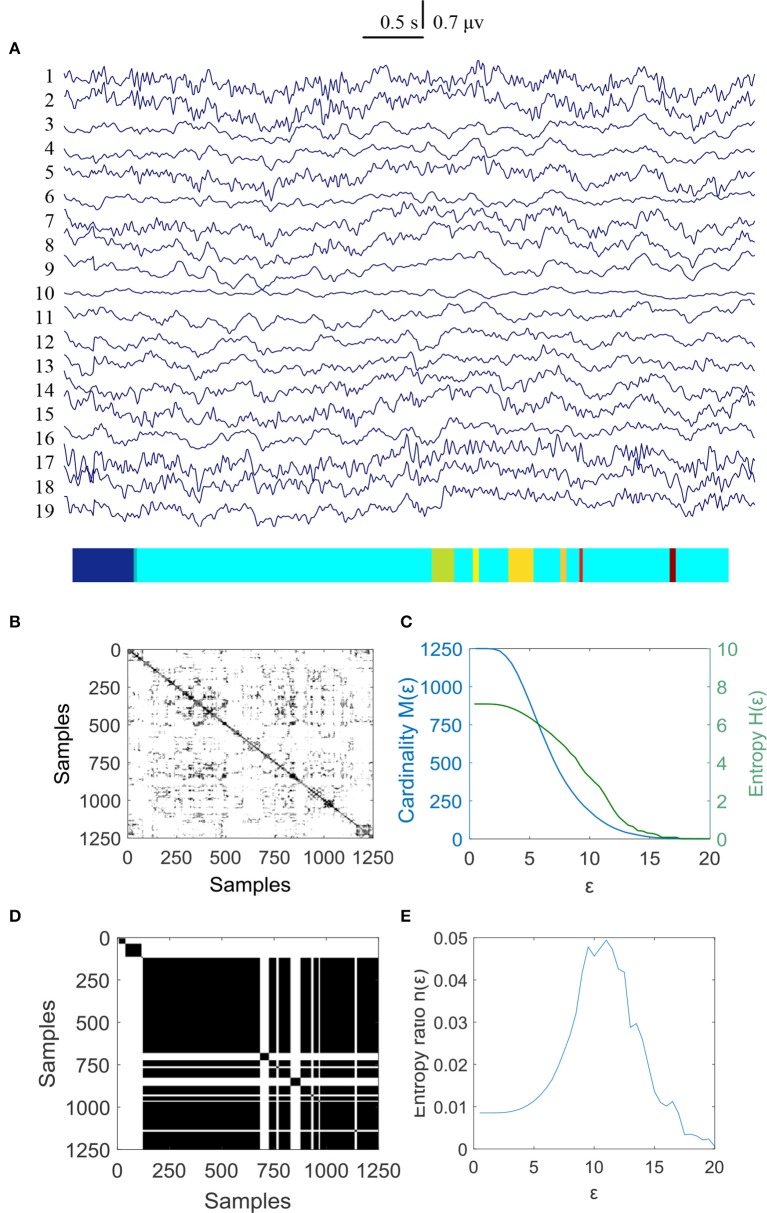
MER analysis of an EEG segment for the controls at the second time. **(A)** The original EEG data from the controls; **(B)** The recurrence plot of **(A)**, with ε = 10.9; **(C)** Change of entropy and cardinality with ε; **(D)** Symbolic recurrence plot; **(E)** Change of entropy ratio with ε (from 0.01 – 20.00 with 0.01 increment).

The same calculating method was used to measure the changes of MER values for the controls and the results were shown in Figures 4, 5. The results showed that the normalized maximum entropy NMER value is 0.1164 for the first EEG data and it is 0.1171 (*p* = 0.6223>0.05) for the second data after 3 weeks, which showed a tiny change. The statistical results are listed in Table 2.

**Table 2:** Statistic results of ε^*^ and NMER values for the controls.

	**Before**	**After**
ε^*^	25.15 ± 16.4993	25.21 ± 15.5150
NMER	0.1164 ± 0.0266	0.1171 ± 0.0378

## Discussion

This paper is the first one describing tDCS-induced changes of EEG complexity in children with ASD. Specifically, the MER algorithm was used and the results showed an obvious increase when the ASD children received 10 tDCS interventions.

As presented in the introduction, several authors have proposed the idea that the neuronal network excitation and inhibition imbalance might be the critical component in the pathogenesis of ASD and severe behavioral deficits in autism have been found arising from elevation in the cellular balance of excitation and inhibition within the neural microcircuitry (Gogolla et al., 2009; Yizhar et al., 2011). For autism, repetitive/restricted behavior is expected to be generated by repetitive/restricted neural network dynamics, which have low entropy than typical development children. Previous research has shown that EEG complexity, as an index for neural information processing and neural connectivity (Sakkalis et al., 2008) is reduced in ASD patients. The presence of reduced EEG complexity was demonstrated in adults with a diagnosis of ASD, supporting the hypothesis that EEG complexity is a sensitive marker for the presence or predisposition of an autistic condition (Catarino et al., 2011).

TDCS research will produce acceptable, non-invasive, safe, quick-acting, and long-lasting treatments. It also can target specific tissues and neural networks with minimal or no deleterious side effects for neurocognitive and behavioral functions. The depolarizing effects of anodal tDCS on neuronal resting membrane potentials and its demonstrated influence on LTP in neuronal circuits provide some account for the observed excitatory effects of anodal stimulation on behavior (Meinzer et al., 2015).

In this study, The MER changed significantly from 13 autism children who received 10 times tDCS modulation. Compared with another waiting group, there was almost no change. Thus, the results helped to reinforce the idea that tDCS applied over the left DLPFC preferentially affects the brain complexity, which can be measured by the alteration of entropy. This change in EEG complexity is specific to the site of stimulation and could be used to target activity as an outcome measure in clinical trials. In fact, tDCS can modulate brain activity in ways that benefit aspects of cognition that are directly related to learning, acquisition, and performance (Coffman et al., 2014). Anodal tDCS intervention over DLPFC could increases the cortical excitability for ASD children and balances the excitation and inhibition of neurons. Previous studies have reported that a single stimulation of anodal tDCS over the left DLPFC resulted in a significantly greater increase in peak alpha frequency measured from the F3 electrode (Amatachaya et al., 2015).

There are some limitations in this study. First, resting-state EEG of ASD children can be influenced by many factors, including EOG and other movements. Second, the stimulation region of DLPFC was not confirmed by neuroimaging but rather by using a slightly less accurate F3 placement of the standard international system. Additionally, sham stimulation was not obtained in the experiment. Despite these limitations, our study demonstrated that anodal tDCS over DLPFC increases EEG complexity. Up to date, there are only symptomatic treatments for autism children may of which suffer from serious side effects. tDCS may be an alternative for ASD patients who are not interested in or decline psychopharmacological treatment. Further research is needed to examine the potential of brain stimulation treatments for ASD and to interpret the mechanisms of these treatments using neuroimaging techniques.

## Author contributions

JK: Writing and data analysis; EC, JH, and ZT: Data recording; XinL: Data analysis; ES and MC: revise; GO: computational analysis; XiaL: Design and revise.

### Conflict of interest statement

The authors declare that the research was conducted in the absence of any commercial or financial relationships that could be construed as a potential conflict of interest.
